# Review of Small Cell Carcinomas of the Prostate

**DOI:** 10.1155/2011/543272

**Published:** 2011-08-04

**Authors:** P. Furtado, M. V. A. Lima, C. Nogueira, M. Franco, F. Tavora

**Affiliations:** ^1^Department of Pathology, Escola Paulista de Medicina Universidade Federal de São Paulo (UNIFESP), Rua Botucatu 740, São Paulo, SP, Brazil; ^2^Presidente Dutra University Hospital, Federal University of Maranhao, São Luis, MA, Brazil; ^3^Departments of Pathology and Urology, Cancer Institute of Ceara, CE, Brazil

## Abstract

Small cell carcinoma of the prostate is a rare neoplasm, with only a few series hitherto reported. A little less than half of the cases are associated with conventional acinar adenocarcinoma, which are usually high grade. Although consensus has not been reached, the majority of patients with small cell neuroendocrine carcinoma of the prostate have advanced disease at diagnosis and disproportionally low PSA levels compared to patients with conventional acinar adenocarcinoma. Treatment consists mainly of chemotherapy associated with surgery. Radiation therapy is reserved for selected cases. This study reviews the most up-to-date information on small cell carcinomas of the prostate.

## 1. Introduction

Small cell neuroendocrine carcinoma of the prostate is a very uncommon type of prostate cancer, which was first described by Wenk et al. [1]. Lacking a specific classification, neuroendocrine tumors of the prostate are usually reported as carcinoid tumors, which are low-grade neuroendocrine carcinomas, and high-grade neuroendocrine carcinoma, which encompasses large neuroendocrine, small cell neuroendocrine carcinoma, and combined tumors, based on their histological and immunophenotypical profile [2–7]. 

Barely more than half of small cell carcinomas arising in the prostate are pure without an associated nonsmall cell component. A large number of cases are detected after androgen ablation therapy for conventional adenocarcinoma. In these situations, conventional acinar adenocarcinoma cells may differentiate along neuroendocrine lines [8].

The importance in recognizing small cell neuroendocrine carcinoma resides in its histological overlap with primary high Gleason-grade tumors of the prostate and its biological behavior, which implies in a different clinical presentation and treatment approach [9, 10]. 

Herein, we review the most up-to-date information on small cell neuroendocrine carcinoma of the prostate, focusing on its histological, immunophenotypical profile as well the most important differential diagnosis. Brief considerations on molecular pathology advances as well as treatment options are also stated.

## 2. Methods

A literature search for small cell neuroendocrine carcinoma of the prostate was performed. Referred articles were selected and reviewed, and data concerning clinical data of cancer occurrence, histological settings, immunohistochemical and molecular profiles, and treatment options are here discussed.

## 3. Discussion

Small cell neuroendocrine carcinoma is rare outside the lung. Approximately 10% cases occur in the prostate, making it one of the most common extrapulmonary sites [3, 6, 8, 9, 11–17]. PSA serum levels can vary from undetectable, especially in cases of conventional acinar tumors with prior hormone treatment, to high levels, with a mean level of 4.0 ng/dL(range 0–1896) in one large series [2, 6, 18, 19]. In this series, the interval between the diagnosis of small cell carcinoma subsequent to one of conventional tumors had a mean of 25 months [6]. Interestingly, in another series focusing on large cell neuroendocrine carcinoma (LCNEC), the interval between initial diagnosis of conventional tumor was higher, with a mean of 4.7 years [2]. Stage at presentation seems also to be higher in cases of tumors with neuroendocrine differentiation that were not subject to hormonal treatment [20]. 

In clinical studies where serum levels of PSA and chromogranin are followed, patients with increasing levels of those markers are diagnosed in an interval of 10 to 30 months, although it is still controversial if serum chromogranin levels independently correlate with prognosis and/or the presence of neuroendocrine differentiation in a given tumor [21–24]. 

**Figure 1:**
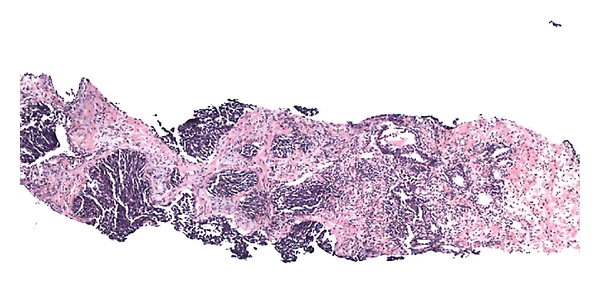
Low power of small cell carcinoma (left) associated with Gleason 7 acinar adenocarcinoma (right).

Histological findings are identical to those tumors arising in extraprostatic sites: in small cell carcinoma, neoplastic cells are arranged mostly in a monomorphic pattern of small round or fusiform cells containing oval or convoluted hyperchromatic nuclei with a salt-and-pepper pattern chromatin, rarely with one or more discernible small nucleoli (Figure 1). Two types of tumor cells can be seen, the classic “oat cell” morphology and also an intermediate cell-type variant which have been described previously in other sites of the body [6]. The classic morphology is characterized by cells only slightly larger than lymphocytes with open chromatin and inconspicuous nucleoli, wherein the intermediate cell type, the tumor cells have more abundant cytoplasm, larger nuclei, and occasional visible nucleoli [6]. 

**Figure 2:**
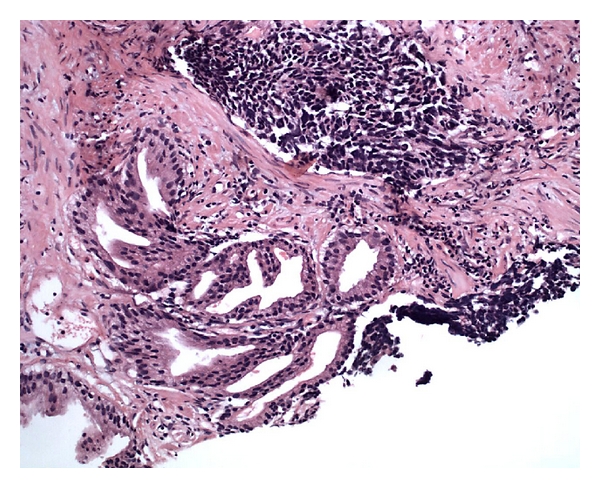
Combined acinar adenocarcinoma and small cell carcinoma diagnosed in a needle biopsy.

**Figure 3:**
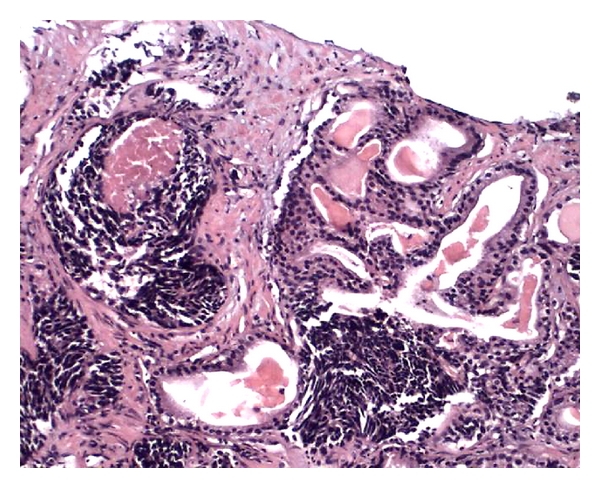
The same case as in Figure 2. Note gradual merging of small cell carcinoma with glands of acinar adenocarcinoma.

**Figure 4:**
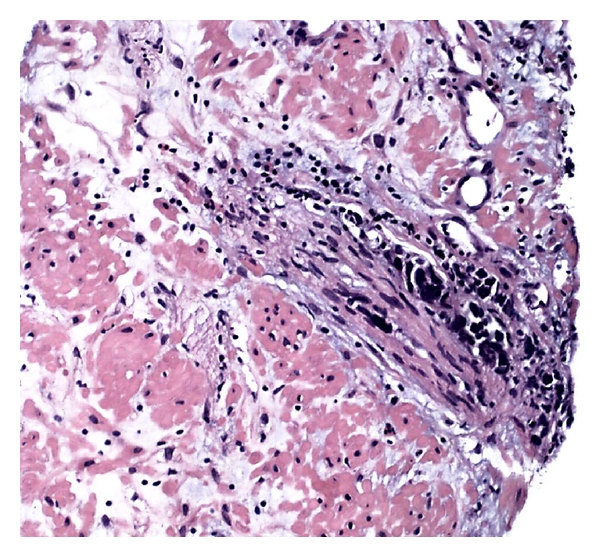
Perineural invasion by the small cell carcinoma component, diagnosed in a needle biopsy.

Pure small cell neuroendocrine carcinomas of the prostate are slightly more common than mixed small cell-adenocarcinomas. The latter occur usually with a high-grade component (Gleason ≥ 8) (Figures 2 and 3) [2, 6, 25, 26]. Cytoplasm is scant. Mitoses are readily discernible and can be numerous. Necrosis is another common histological finding but is usually not extensive. Perineural invasion is also common (Figure 4). Larger atypical cells, formation of true rosettes or pseudorosettes, and a large clear and vacuolated cytoplasm are also described. Another spectrum of neuroendocrine differentiation encompasses Paneth cell-like change. This phenomenon was reported by Weaver et al. and is characterized by the presence of small eosinophilic cytoplasmatic granules resembling intestinal Paneth cells in prostate cancer. Its true neuroendocrine origin is confirmed by immunohistochemical and electron microscopy studies [27].

For the surgical pathologist, the most critical and common issue concerning the diagnosis of a small cell neuroendocrine carcinoma is its confusion with a poorly differentiated acinar adenocarcinoma (Gleason 5), notably those with a solid pattern without gland formation and central necrosis in a small focus on needle biopsies. Indeed, misdiagnosing small cell carcinomas as high-grade acinar adenocarcinoma seems to occur commonly. Studies reveal a 0.5–2% incidence of small cell carcinoma in patients diagnosed in biopsies as opposed to a 10%–20% figure in autopsies cases [28, 29].

**Figure 5:**
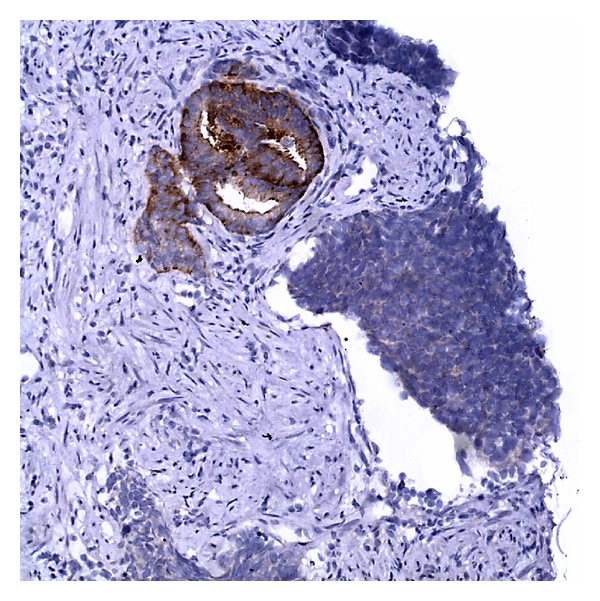
PSA immunostain showing strong positivity in benign prostate glands, whereas the neuroendocrine tumor is faint to absent.

**Figure 6:**
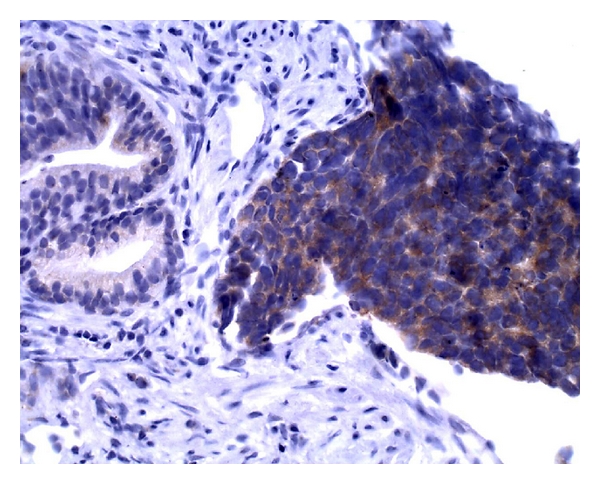
Small cell carcinoma with strong positivity for chromogranin immunostain.

Although not required for the diagnosis of small cell carcinoma, immunohistochemical studies may be helpful (Table 1). A comprehensive immunohistochemical panel to differentiate small cell carcinoma from poorly differentiated adenocarcinoma includes PSA, PSAP, P501s, and neuroendocrine markers, CD 56 being the most sensitive for small cell carcinoma (Figures 5 and 6). TTF-1 can be positive in up to half of small cell carcinomas and is not found in the poorly differentiated adenocarcinomas [6, 17]. Most small cell carcinomas are negative for the aforementioned prostate markers (PSA, PSAP, and P501S), with some rare cases showing focal positivity, while poorly differentiated adenocarcinomas are usually diffusely positive for the same antibodies. Expression of neuroendocrine markers can be seen in conventional acinar adenocarcinomas, and the diagnosis of neuroendocrine carcinomas should rely in both immunohistochemical profile and light microscopic morphology. 

Pulmonary small cell carcinomas are aggressive neoplasms commonly in advanced stages at diagnosis. PSA serum levels are not commonly elevated in primary small cell carcinomas of the prostate, and its levels are not helpful in separate metastatic lung disease from prostate small cell carcinoma. Immunohistochemistry can be helpful in distinguishing them, as small cell carcinomas can be positive (even focally) for at least one prostatic marker (PSA, PSMA, PSAP, or P501s) which are not expressed in lung tumors [36]. CD44, a cell-surface molecule proposed to identify cancer stem/progenitor cells in prostate cancer, has been demonstrated to be highly specific of small cell carcinoma of the prostate, when compared to conventional acinar adenocarcinoma or small cell carcinomas of other sites [10, 30]. 

The recent discoveries of the TMPRSS2-*ERG* rearrangement in subset of prostate cancer, with prevalence between 40–70% of all tumors, raised the question of the presence of this genetic aberration in more aggressive forms of prostate tumors. Two recent papers have addressed the issue of the translocation in small cell carcinomas of the prostate. Guo et al. [37] evaluated the TMPRSS2-*ERG* gene fusion in 12 small cell carcinomas of the prostate with small cell carcinomas of the bladder and lung as control, by fluorescent in situ hybridization (FISH), and found the aberration in about 67% of the cases and in none of the controls. In a similar study, Lotan et al. [38] also found the *ERG* translocations in more than 45% of small cell carcinoma of the prostate, and in cases where the acinar component was also available for analysis, there was concordance for the presence/absence of *ERG* gene rearrangement between the different subtypes. These findings strongly suggest a common pathway of genesis of conventional acinar and also small cell carcinoma of the prostate. 

More recent data on molecular characterization of small cell carcinoma of the prostate reported by Tai et al. [39] have shown specific association of those cases with PC3, one cell line related to prostatic carcinoma. Indeed, those cells are immunohistochemically characterized by the expression of CD44, a stem cell marker commonly reported and believed to be more specific for small cell carcinoma of the prostate. On the contrary, conventional adenocarcinoma do not show CD44 positivity and have expression of PSA and androgen receptors like LNCaP, another known cell line associated with prostatic carcinoma [39].

The treatment of small cell carcinoma of the prostate includes a multimodality approach with chemotherapy as the mainstay of treatment, and radiation as supplemental for local control or for palliation. However, no uniform treatment being clearly established. Regimens that include gemcitabine, docetaxel and carboplatin, or cisplatin have been attempted with variable success [40–42]. Radiotherapy is also used, since patients with a small carcinoma diagnosis are not common candidates for surgical treatment [10, 14, 43, 44]. However, primary surgery was the most important prognostic factor for prolonged survival in one study [45]. Neuroendocrine differentiation may play an important role in the development of androgen resistance [14, 41], and advanced prostatic carcinomas with pure or partial neuroendocrine differentiation have a median survival of only 10 months. However, a case of mixed conventional acinar adenocarcinoma and small cell neuroendocrine carcinoma recently reported by Brammer et al. treated with concomitant hormonal and chemotherapy showed complete remission of disease 36 months after the initial diagnosis [46].

Another potential target for the treatment of small cell neuroendocrine carcinoma is the relaxin receptor RXFP1. Relaxin is a small peptide hormone expressed in several cancers such as those of endocrine origin. Its receptor, RXFP1 (a G-protein-coupled receptor), is expressed in androgen receptors' positive and negative cancers, as well as in prostate germ cells. In PC3 prostate cancer cell lines, which include small cell neuroendocrine carcinoma, treatment of RXFP1 showed significant reduction of tumor size, decrease in cell proliferation and metastatic disease, and increased apoptosis [47].

## Figures and Tables

**Table 1:** Immunohistochemical findings of small cell carcinoma of the prostate compared with conventional high-grade adenocarcinomas [6, 8, 10, 17, 20, 30–35].

Antibody	Small cell carcinoma	Poorly differentiated
	(approximate	adenocarcinoma
	percentage of	(approximate percentage
	positivity)	of positivity)
Cytokeratin	(94%)	+ (70%)
Cytokeratin high	(35%) −/+	− (0-33%)
molecular weight		
CAM 5.2	(72%)	+ (90%)
CK 7	(39%) −/+	−/+ (30%)
CK 20	(11%) −/+	−/+ (10%)
PSA	(24%) −/+	++ (85%)
PSMA	(20%) −/+	++ (90%)
PSAP	(22%) −/+	++ (95%)
P501s	(25%) −/+	++ (90%)
p63	(40%) −/+	−−/+ (15%)
TTF1	(83%) +/−	− (10%)
CD 56	(92%) +	− (10%)
Chromogranin	(80%) +	− (10%)
Synaptophysin	(85%) +	−/+ (13%)
CD44	(60-96%) ++	− (5%)
